# Pulmonary Histoplasmosis With Superimposed Pneumocystis Pneumonia

**DOI:** 10.7759/cureus.43152

**Published:** 2023-08-08

**Authors:** Mohammad Usman, Ashley C Calise, Arsh N Patel, Austin Huang, Laurence Stolzenberg, Mohammad Ibrahim, Navneet Kaur

**Affiliations:** 1 Psychiatry, Alabama College of Osteopathic Medicine, Dothan, USA; 2 General Surgery, Alabama College of Osteopathic Medicine, Dothan, USA; 3 Orthopaedic Surgery, Alabama College of Osteopathic Medicine, Dothan, USA; 4 Neurology, Alabama College of Osteopathic Medicine, Dothan, USA; 5 Health Sciences, University of Central Florida, Orlando, USA; 6 Internal Medicine, North Alabama Medical Center, Florence, USA

**Keywords:** pneumonia, alabama, immunocompromised, hiv/aids, pneumocysticosis, histoplasmosis

## Abstract

Histoplasmosis is a fungal infection that, if left untreated, can result in very serious health outcomes, especially in patient populations that are immunocompromised. While the manifestations of the disease are very diverse and highly dependent on the individual health conditions of the patient, in severe cases, it can lead to serious pneumonia, acute respiratory distress syndrome, and death if rapid medical intervention is not performed. Here, we present the case of a patient with acquired immunodeficiency syndrome who suffered from histoplasmosis pneumonia with suspected superimposed *Pneumocystis* pneumonia. The patient rapidly decompensated shortly after admission to the hospital; he presented just one week after being discharged of a similar infection. After being transferred to the intensive care unit (ICU), aggressive intervention stabilized the patient’s condition enough for him to be discharged several days later. We hope the unique circumstances of this patient’s hospital stay can guide clinicians in managing serious infections in immunocompromised patients.

## Introduction

*Histoplasma capsulatum* (*H. capsulatum*) is a commonly found endemic mycosis that is responsible for histoplasmosis, a rare but potentially fatal infection. The causative pathogen is found throughout the world but is most commonly located in North America. Within the United States, the most common locations where it is found are the Ohio and Mississippi River valleys. It is estimated that between 60% and 90% of people living in those areas have been exposed to *H. capsulatum* at some point [1,2]. Infection by histoplasmosis is more common in immunocompromised patients, but it can also be found in immunocompetent patients. Infections are usually mild and limited to the pulmonary systems in most patients. These cases tend to resolve on their own without medical interventions. However, in severe cases, serious life-threatening complications can develop, especially if the infection disseminates to other organ systems [1].

*H. capsulatum* is commonly found in areas that have been exposed to bird or bat droppings, and as such, exposure to areas where such droppings would be common, such as chicken coops and caves, drastically increases the risk of exposure. In addition, activity that disturbs soil, such as construction, has also been known to increase the risk of exposure to the pathogen [3-5]. It is important to note that, of the endemic mycosis found in the United States, histoplasmosis is the most common cause of hospitalizations and should always be on the mind of the discerning clinician when encountering a patient with a constellation of symptoms, such as fevers, chills, myalgias, cough, and chest pain [6,7].

Disseminated histoplasmosis is a serious and potentially fatal complication of infection and is most associated with serious immunocompromised states [8,9]. It is incredibly urgent to identify and begin treatments for patients who are at higher likelihood of infection, as untreated dissemination in immunocompromised patients is almost always fatal without urgent and aggressive intervention [4,7]. The treatment for disseminated histoplasmosis is generally induction of broad-spectrum anti-fungal medications, such as liposomal amphotericin B and supportive care [9,10].

## Case presentation

**Figure 1:**
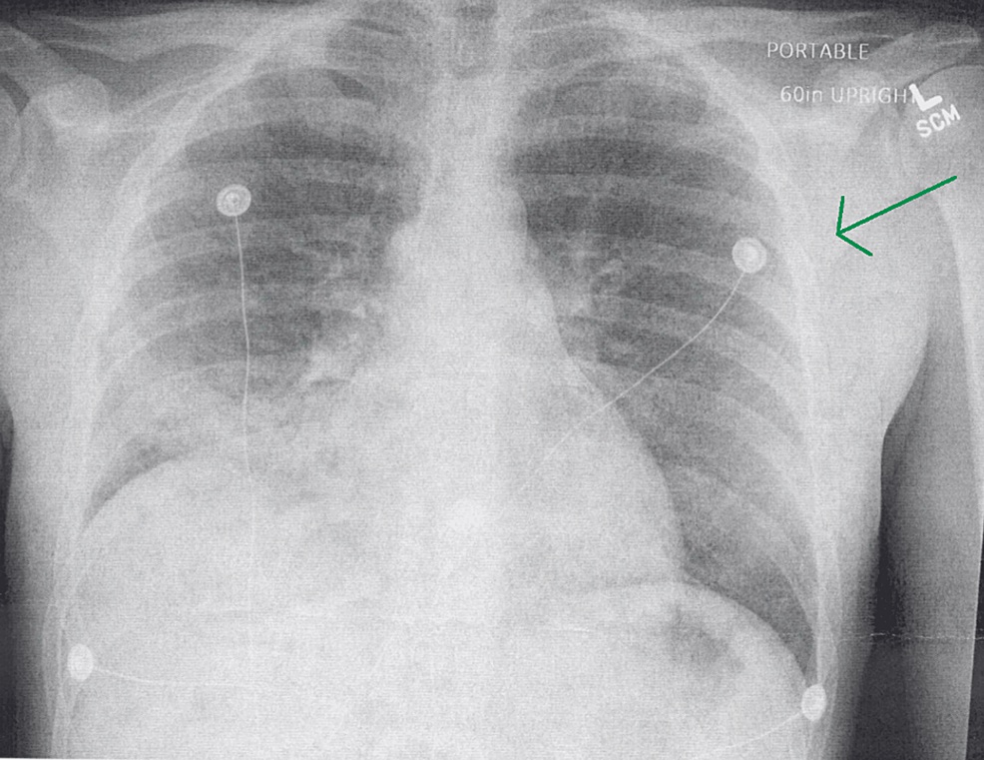
Anterior-posterior chest X-ray from initial visit

The patient in this case was a 29-year-old male who presented to the emergency department complaining of fever, shortness of breath, and pleuritic chest pain. The patient had previously been admitted to the same hospital for the exact same complaint and had subsequently been discharged one week prior to his current presentation. On his prior admission, a thorough investigation revealed elevated lactate dehydrogenase (LDH) and β-glucan of greater than 500 pg/mL, leading the clinicians in charge of his care to diagnose him with *Pneumocystis *pneumonia (Figure 1).

At this time, he also tested positive for *Histoplasma* antibodies at a high titer, although his urine antigen was negative. He also tested positive for HIV at this time. This lead the clinicians in charge of his care to conclude that there was a serious potential risk of disseminated histoplasmosis, which could be very rapidly fatal if left untreated. However, after consultation with infectious disease (ID), it was their belief that disseminated histoplasmosis was not necessarily the most likely source of his illness. They instead concluded that there was likely serious pulmonary involvement of histoplasmosis. He was treated in the inpatient unit for 10 days, during which time he was given ceftriaxone for seven days, trimethoprim-sulfamethoxazole for 21 days, and prednisone, as well as his antiretroviral therapy (ART) before he was discharged home with instructions to follow up with a local clinic. His medication regimen upon discharge was ceftriaxone for seven days, trimethoprim-sulfamethoxazole 800/160 mg every eight hours for an additional 14 days to complete 21 days in total, and itraconazole 200 mg three times a day for three days, followed by 200 mg twice a day. He was also given albuterol 90 mcg every four hours as needed for shortness of breath, hydrocodone-acetaminophen 5/325 mg as needed every six hours for pain for five days, and lastly prednisone 40 mg twice a day for five days followed by a taper of 40 mg a day for five days, further followed by 20 mg a day for 11 days for hypoxia. The patient was also continued on his preexisting ART and other medications, with instructions for close follow-up if his symptoms returned.

On the return admission, the patient presented with severe chest pain when taking deep breaths that worsened with sitting down and improved when walking. He also claimed to have nightly fevers reaching 106 °C with associated symptoms of chills and sweating. He reported the reason for his return to the emergency room was because on follow-up with his outpatient provider, he was encouraged to return to the hospital for evaluation and treatment. The patient also reported fatigue, generalized weakness, shortness of breath on ambulation, frontal headaches, generalized abdominal pain, and a dry cough with occasional, white-tinged sputum expectorated. The patient’s medical history was significant for recently diagnosed HIV and severe candidal esophagitis approximately one and a half months prior. On admission, the patient’s medication list included medications listed previously and lorazepam 1 mg twice a day and tramadol 50 mg three times a day as needed. Social history was significant for a pack-a-day smoking history for the previous 10 years. The patient denied using any alcohol and IV drugs, but he reported marijuana use. The patient lived at home with his wife and children. The patient met all independent activities of daily living and was employed as a welder. When questioned regarding previous or current high-risk behaviors, the patient was unable to identify the source of his HIV infection.

Initial physical exam revealed an alert and oriented African American male, who was able to answer all questions and follow instructions. He was placed on 2 L low-flow nasal cannula for oxygen secondary to his mild respiratory distress. His mucous membranes were dry. He exhibited pale conjunctiva and anicteric sclera. Crackles and rhonchi were appreciated over most lung fields bilaterally. His heart examination was normal. However, his abdominal examination revealed mild diffuse tenderness, without distention or organomegaly, and with normoactive bowel sounds. His initial vital signs and blood work are shown in Table 1 and Table 2.

**Table 1:** Initial vital signs on second admission

Vital sign	Value	Reference range
Temperature	97.5˚F	97.8-99.1˚F
Pulse	113 beats per minute (bpm)	60-100 bpm
Respiratory rate	18 breaths per minute (bpm)	12-18 bpm
Blood pressure	108/72 mmHg	90/60 - 120/80 mmHg
Pulse oximetry	99% on room air	95-100%

**Table 2:** Initial lab values on second admission WBC: white blood cell; RBC: red blood cell; MCV: mean corpuscular volume; MCHC: mean corpuscular hemoglobin concentration; RDW: red cell distribution width; eGFR: estimated glomerular filtration rate; AST: aspartate transaminase; ALT: alanine transaminase; ALP: alkaline phosphatase

Laboratory	Value	Reference range
WBC	5.9 x 10^9 ^cells/L	5.0-10 x 10^9 ^cells/L
RBC	3.46 x 10^12 ^/L	4.7 - 6.2 x 10^12 ^/L
Hemoglobin	10.7 g/dL	14 – 17 g/dL
Hematocrit	31.8%	42-52%
MCV	91.9 mm^3^	82-98 mm^3^
MCHC	33.7 g/dL	32-36 g/dL
RDW	17.9%	11.8-14.5%
Platelets	227,000/mm^3^	150,000-400,000/mm^3^
Neutrophils	81.4%	54-62%
Lymphocytes	12.0%	25-33%
Monocytes	6.0%	3.0-7.0%
Eosinophils	0.3%	1.0-3.0%
Basophils	0.3%	0.0-0.75%
Sodium	120 mEq/L	135-145 mEq/L
Potassium	4.8 mEq/L	3.5-5.0 mEq/L
Chloride	98 mEq/L	95-105 mEq/L
CO_2_	23 mmHg	33-45 mmHg
Anion gap	8 mEq/L	4-12 mEq/L
Blood urea nitrogen	15 mg/dL	3.57—7.14 mg/dL
Creatinine	0.7 mg/dL	0.7 to 1.3 mg/dL
eGFR	128 mL/min/1.73^2^	> 60 mL/min/1.73^2^
Glucose	95 mg/dL	< 200 mg/dL
Osmolality	251 mOsm/kg H_2_O	285-295 mOsm/kg H_2_O
Calcium	8.5 mg/dL	9.0 - 10.5 mg/dL
AST	32 units/L	10 - 40 units/L
ALT	30 units/L	10 - 55 units/L
ALP	61 units/L	44 - 147 IU/L
Protein	6.3 g/dL	5.4 - 8.3 g/dL
Albumin	2.8 g/dL	3.5 - 5.0 g/dL
Globulin	3.5 g/dL	2.0 - 3.5 g/dL
Albumin/globulin ratio	0.8	1.5 - 2.5

**Figure 2:**
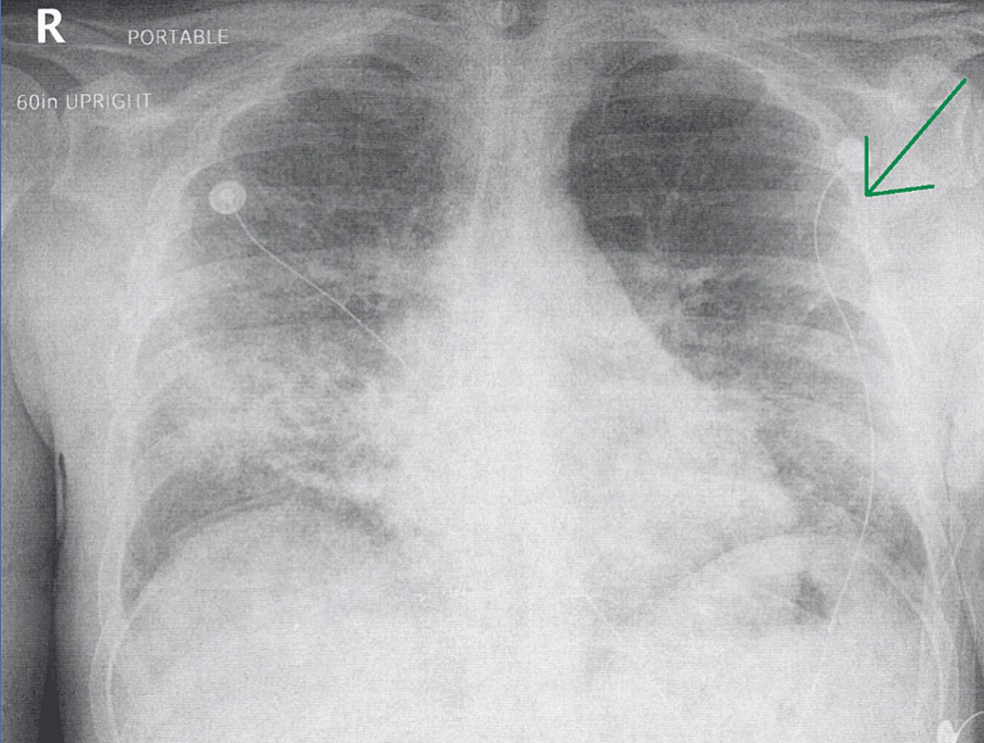
Anterior-posterior chest X-ray on repeat admission

**Figure 3:**
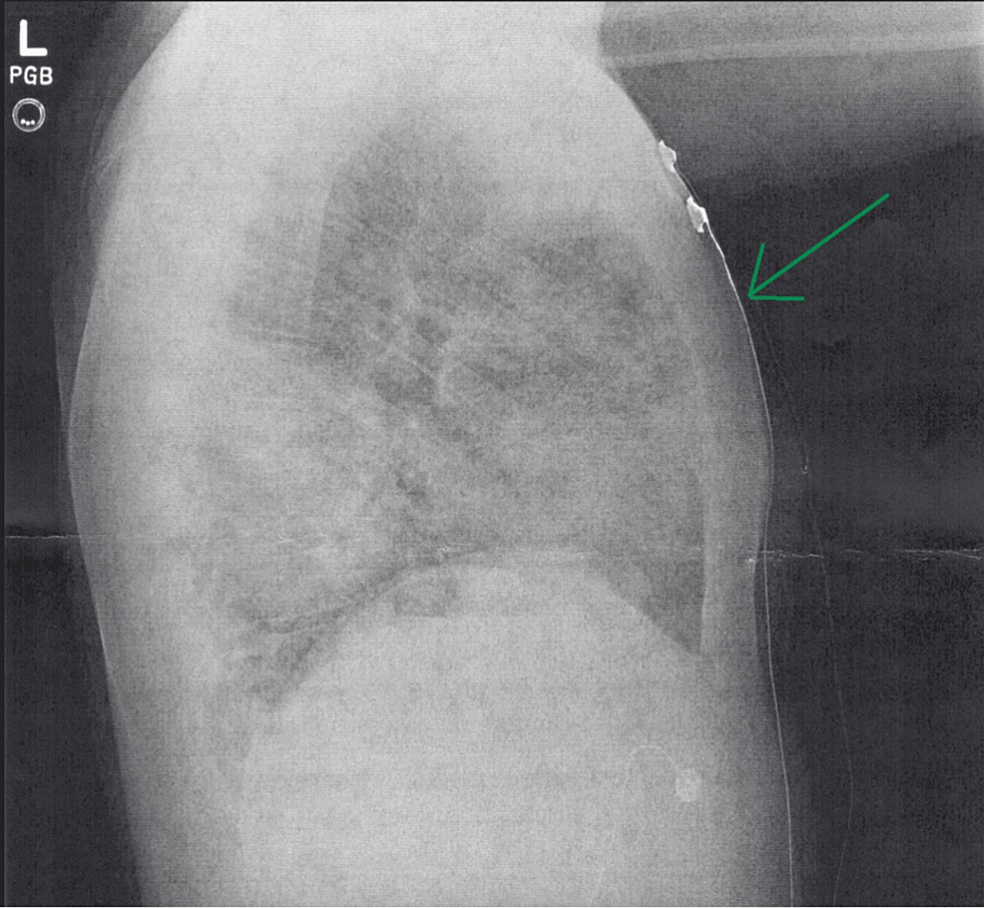
Lateral chest X-ray on repeat admission

Initial imaging revealed worsened diffuse opacities in the lung, consistent with pneumonia (Figures 2, 3). Given the patient’s recent admission to the hospital, it was also considered that the patient’s pneumonia could potentially have been caused by a hospital-acquired pathogen superimposed on the preexisting pneumonia. Faced with the constellation of symptoms and subsequent investigation, the patient was diagnosed with acute respiratory failure with hypoxia and hypercapnia and was admitted to the floor. Shortly after his admission, his condition rapidly worsened. It was noticed on reexamination that the patient’s breathing was becoming more labored, although his mentation remained appropriate. An arterial blood gas (ABG) was performed, which showed a pH of 6.915, pCO_2_ of 84 mmHg, pO_2_ of 121 mmHg, and a bicarbonate of 17 mEq/L.

Given the serious nature of his results and the potential for rapid decompensation, it was decided to transfer the patient from the floor to the intensive care unit (ICU). At the same time as the transfer, the patient’s temperature spiked to 102°F and his heart rate elevated to 147 bpm. Given that the ABG showed serious respiratory acidosis and that the patient was starting to use accessory muscles of respiration, he was started on bilevel-positive airway pressure (BiPap). It was decided as well to consult ID in order to best formulate the treatment plan. ID recommended starting the patient on empiric treatment and prescribed cefepime 1000 mg every eight hours, metronidazole 100 ml every eight hours, vancomycin 250 ml, and piperacillin-tazobactam 4.5 grams every six hours. They also recommended the continuation of the anti-fungal regimen previously prescribed, which was itraconazole 200 mg. In addition to the antibiotics, he was also started on enoxaparin 20 mg for a mildly elevated D-dimer of 0.91 mg/L fibrinogen equivalent units (FEU), which has a normal range of less than 0.5 mg/L FEU. The patient was tested for COVID-19, influenza, and *Streptococcus pneumoniae*, all with negative results. Cluster of differentiation 4 (CD4) lymphocyte testing was conducted, resulting in a CD4 count of 138 cells/mm^3^. This is significantly lower than the normal range of 500-1500 cells/mm^3^, and any number less than 200 cells/mm^3^ is consistent with a diagnosis of AIDS.

The patient was then treated for several days in the ICU with the aforementioned treatment and slowly weaned off BiPap onto supplemental oxygen by the subsequent day after admission. Cultures of the nares were negative for methicillin-resistant *Staphylococcus aureus,* and based on the clinical improvement and negative lab results, it was decided to discontinue the vancomycin and piperacillin-tazobactam. Sputum and blood cultures were also negative at this time for any other pathogens; fungal cultures were pending and LDH levels continued to decrease. Two days after admission and initiating treatment, the patient’s clinical condition was greatly improved, as he was afebrile and no longer in acute respiratory distress. Four days after admission, the patient had recovered sufficiently for discharge. His medications on discharge were itraconazole 200 mg twice a day for 10 days orally, prednisone 70 mg once a day for 10 days, and trimethoprim-sulfamethoxazole 800-160 mg three times a day for 30 days, as well as his existing medications. Upon discharge, physical exam showed greatly improved pulmonary function, and a resolution of his admitting symptoms and was advised for close follow-up. As of writing, the patient is continuing to recover.

## Discussion

This case of a patient who was severely decompensated shortly after admission in what was a short but serious clinical course is something that can and should be a learning experience for clinicians. HIV infection with subsequent presentation of AIDS is something that should be in the back of mind for almost any patient encounters, even when the patient has a low risk of being infected [11]. This patient was only diagnosed with HIV after starting to suffer serious health complications and, as a result, was only able to get adequate medical attention after already experiencing dangerous health issues. It is for this reason that the United States Preventive Services Task Force recommends screening all patients aged 16 to 65 for undiagnosed HIV infection [12]. Had the diagnosis of HIV been established during routine screening, before the development of AIDS in this patient, he likely would have been able to start ART early enough to prevent his illness.

Clinicians should also consider less common causes of serious infections, such as histoplasmosis. While histoplasmosis and* Pneumocystis* are not exceptionally rare illnesses, especially in immunocompromised patient populations [1,3], they are certainly not the first pathogens that would immediately come to mind. It, therefore, is important to keep fungal infections in the differential diagnosis as the potential harm of not assessing for the possibility of a fungal pathogen can lead to potentially disastrous outcomes. Moreover, clinicians should make more of an effort on patient education about the diseases they face and the symptoms that might be indicative of a more serious outcome. This patient was seen and treated less than one week prior to his readmission, and he was in a much worse state at this time. While his initial presentation was not nearly as severe and his treatment on his initial visit was excellent, more work could have been done to ensure a safe transition from the inpatient to community settings. This patient only returned to the hospital setting because of his follow-up with an outpatient clinic, and considering his rapid decompensation after admission, it was just in the nick of time. His constellation of symptoms [7,13] in the previous week were severe enough that he should have been educated as to return to the hospital. This example is one reason why physicians should take great care in making sure our education to patients is clear, concise, and thorough in order to ensure that patients are given the best-quality medical care available.

## Conclusions

We hope that this case can guide clinicians in the management of seriously ill patients who may not be presenting with the usual culprits of serious decomposition. This case should also be able to provide guidance to physicians treating patients with serious HIV/AIDS and other immunocompromised conditions, on both the likely source of infection and the best course of action to treat such serious illnesses. It is always a good idea to also consider the fact that any patient encountered, even in contexts not related to serious diseases, could potentially be immunocompromised by infections, autoimmune conditions, or other causes. As such, utmost care should be taken to ensure that patients are screened in accordance with evidence-based guidelines, in the hope of preventing serious infections and illnesses ever taking root in the first place.
